# Sensory Phenomenon Assessment Scale: a new tool for assessment of tic-associated sensations

**DOI:** 10.3389/fpsyt.2024.1387417

**Published:** 2024-06-24

**Authors:** Xianbin Wang, Yanlin Li, Liping Yu, Hui Xu, Anyi Zhang, Wenyan Zhang, Zhongliang Jiang, Yonghua Cui, Ying Li

**Affiliations:** ^1^ Department of Psychiatry, Beijing Children’s Hospital, Capital Medical University, National Center for Children Healthy, Beijing, China; ^2^ Peking University Sixth Hospital, Peking University Institute of Mental Health, National Health Commission (NHC) Key Laboratory of Mental Health (Peking University), Beijing, China; ^3^ Big Data Center, Beijing Children’s Hospital, Capital Medical University, National Center for Children’s Health, Beijing, China; ^4^ Department of Psychosomatic Medicine, Beijing Children’s Hospital, Capital Medical University, National Center for Children Healthy, Beijing, China

**Keywords:** tic disorder, premonitory urge, Sensory Phenomenon Assessment Scale, reliability, validity

## Abstract

**Background:**

Sensory symptoms linked to tic disorder (TD) are challenging to quantify via self- or parent-reported measures. The current study aimed to develop a novel observer-rated semi-structured interview, namely, the Sensory Phenomenon Assessment Scale (SPAS), to aid clinical evaluation on symptoms of TD among children.

**Methods:**

To test its psychometric properties, tic, premonitory urge (PU), and obsessive–compulsive symptoms (OCS) were also assessed in 223 children via the Yale Global Tic Severity Scale (YGTSS), the Premonitory Urge for Tic Scale (PUTS), and the Children’s Yale–Brown Obsessive–Compulsive Scale (CY-BOCS). Factor analysis and internal consistency test were carried out using data from TD-diagnosed individuals.

**Results:**

Good internal consistency and test–retest reliability were observed. Criterion validity was established by significant correlations between the PUTS, the YGTSS, the CY-BOCS, and scores of the SPAS. Factor analyses supported a single-factor model of the SPAS, in which the five items each showed a factor loading above 0.6.

**Conclusion:**

This study demonstrated that the SPAS is reliable and valid and, thus, can serve as a good and concise measure of clinical symptoms among children and adolescents with TD.

## Introduction

Tic disorder (TD) is a neurodevelopmental disorder commonly found in children and adolescents (1). Premonitory urge (PU) can occur before, during, or after the onset of tic symptoms. Typical PU symptoms include itchiness, pressure, or a sense of incompleteness (2, 3). Several studies (2, 4) have shown that PU is prevalent in patients with TD, especially those with Tourette syndrome (TS). The sensations are also very salient, as many patients with TS describe the PU as more distressing than the tics themselves (5). Recent behavioral models suggest that PU is the cognition-behavioral basis for tic symptoms. After the onset of the tic symptoms, the individual is relieved from the pain of PU, even if sometimes temporarily (5, 6). Based on cross-sectional data (7, 8), a close positive correlation between severity of PU and tic symptoms in TD individuals should be noticed, which also implies that a higher degree of PU predicts more severe tics. These high correlations of theirs may derive from similar neural mechanisms; for example, both motor tic and PU production are correlated with right insula (9) and cingulate cortex volume (10). PU is related to the production, duration, and inhibitory control of tics (11, 12). Fostering awareness and understanding of PU stands as the foundational and pivotal stage in a well-established behavioral intervention for treating TD, Habit Reversal Training (HRT) (13, 14), for example.

Assessing PU objectively holds significance in the examination of TD. Methods devised for evaluating PU encompass both neuropsychological paradigms (11) and assessment scales (15–20). Neuropsychological paradigms were discouraged in clinical evaluation due to their complexity and high demand for equipment. Validated scales for measuring PU include the Premonitory Urges for Tic Disorders Scale (PUTS) (15), its adapted version Premonitory Urges for Tic Disorders Scale-Revised (PUTS-R) (16), Individualized Premonitory Urge for Tics Scale (I-PUTS) (17), Sensory Processing and Self-Regulation Checklist (SPSRC) (18), Rumination and Awareness Scale for Tic-Associated Sensations (RASTS) (19), and University of Sao Paulo Sensory Phenomena Scale (USP-SPS) (20). Among these tools, the PUTS (15) stands out as the most widely used, with translations and validations available in numerous languages (21–23). The majority of clinical studies (4, 24) employed the PUTS to gauge the intensity of PU. The PUTS was developed by Professor Woods (15) and first published in 2005. This self-rating scale has a total of nine items and mainly focuses on the number and frequency of PU. In an earlier study, the reliability and validity of the PUTS (23) have been verified in a Chinese setting by our research team.

However, issues that have not been addressed exist within the current scales. Symptom-related tensity and functional impairment are not fully assessed, while reliability and validity of self-report scales are less solid for children under the age of 10 (15, 25). Therefore, a new type of observer-rated scale is in need to provide more accurate and comprehensive assessment.

The current study aimed to develop and validate a new observer-rated semi-structured interview, namely, the Sensory Phenomenon Assessment Scale (SPAS). Drawing on the latest research on children’s mental health and behavior, we strived to build a tool that accurately reflects the complexity of TD children’s PU symptoms and is suitable for clinical practice. We hypothesized that the new instrument would have good reliability and validity and would be suitable for assessing tic-related sensory symptoms.

## Methods

### Participants

Participants were recruited from the outpatient clinic of Beijing Children’s Hospital from 1 May 2022 to 30 April 2023. Inclusion criteria were as follows (1): patients aged 6–17 years (2); patients who had TD diagnosed by a child psychiatrist according to the Diagnostic and Statistical Manual of Mental Disorders-5 (DSM-5); and (3) patients experiencing PUs (with a total PUTS score exceeding 12). Exclusion criteria were as follows: (1) patients with traumatic brain injury, epilepsy, or intracranial tumors; (2) patients who had comorbid mental disorders; and (3) patients with difficulty communicating.

Informed consent was obtained from each patient and their main caregiver. The procedure of the current study was approved by the Ethics Committee of Beijing Children’s Hospital (approval number: [2023]-E-105-R).

### Measures

The Sensory Phenomenon Assessment Scale (SPAS) was developed by the Delphi method. The final version of the SPAS consisted of two parts with 13 items. The first part was the observer-described “symptom list”, which contained eight items (itch, sense of suffocation, pressure, sense of energy release, sense of energy tension, sense of uncompletion, indescribable discomfort, and other). The second part is “severity” and contains five items, namely, number, frequency, tensity, degree of transformation, and functional impairment. Each item was scored on a six-point scale from 0 to 5, with higher scores indicating severer symptoms. For more details, see **Appendix 1** (Development of SPAS), **Appendix 2** (Final version of SPAS), and **Appendix 3** (SPAS User Manual).

The Yale Global Tic Severity Scale (YGTSS) mainly consists of three parts. The first part is a tic inquiry item. In the second part, the number, frequency, intensity, complexity, and interference of tics were scored. Each aspect was scored from 0 to 5, and the maximum total score was 50. The final section is the overall impairment score, with a maximum score of 50. Higher YGTSS score indicates more serious tic symptoms. The reliability and validity of the scale have been verified in the Chinese-Taiwanese population (26). In addition, we had revisited the structure of this scale in a sample of Chinese children with TDs (27).

The PUTS consists of nine items, each item has five scales with a 0 to 4 score scale (a score of 0 = “none”, 1 = ‘‘not at all true,’’ 2 = ‘‘a little true,’’ 3 = ‘‘pretty much true,’’ and 4 = ‘‘very much true”). Score ranges from 9 to 36. Nine items were used to measure the frequency of sensory symptoms of a different nature, the frequency of sensory transformation into tics, and whether sensory phenomena persisted after tics. The reliability and validity of this scale had been validated in a Chinese population (23).

The Children’s Yale–Brown Obsessive–Compulsive Scale (CY-BOCS) (28) is a 10-item, clinician-rated, semi-structured scale designed to assess the symptom severity of OCD during a subject’s previous week. Each item is rated by a five-point Likert scale (0–4). The total score ranges from 0 to 40. The CY-BOCS consists of two dimensions: obsessive thoughts and compulsive behaviors. Our team has verified the reliability and validity of this scale in the Chinese population (29).

All patients’ severity of tics was evaluated using the SPAS, YGTSS, and CY-BOCS by two experienced child psychiatrists (Yanlin Li and Xianbin Wang). The Pearson correlation coefficient of consistency was 0.91. All the patients and their parents were then asked to fill in the PUTS.

### Statistical analysis

To verify the reliability of the SPAS, we calculated the internal consistency (Cronbach’s α) on all five items of part 2 (“severity of sensory phenomena”), because part 1 (“symptom list”) was not scoring. A split-half reliability analysis was performed on odd and even items and the Spearman–Brown coefficient was computed. Dozens of participants were selected from mild tic patients, who required only clinical observation (no medication or other intervention), and repeated the test 1 month later to calculate the test–retest reliability.

Validity test included criterion-related validity based on the PUTS, YGTSS, and CY-BOCS and exploratory factor analysis (EFA). Correlation coefficients between the SPAS and the PUTS, YGTSS, and CY-BOCS were calculated, respectively. Factor loadings of each item were estimated by EFA.

All the above calculations were performed separately on a sample younger than 10 years of age (group 1), and a sample of age greater than or equal to 10 (group 2) used IBM SPSS Statistics 19.

## Results

### Demographic characteristics and clinical profiles

A total of 223 children and adolescents with TD (187 male and 36 female participants) were enrolled in the testing sample according to inclusion and exclusion criteria. All continuous variables, except for the CY-BOCS and its two subscales, exhibited a normal distribution. The mean age was 10.01 years with a standard deviation of 2.552. The mean SPAS score was 7.94 with a standard deviation of 5.268, while the mean PUTS score was 17.55 with a standard deviation of 3.461. Additionally, the mean YGTSS total score (total score of motor tic, vocal tic, and impairment of tic) was 25.39 with a standard deviation of 10.928, and the CY-BOCS had a median of 2, with an interquartile range of 0–8. See **Table 1** for details.

**Table 1:** The clinical characteristics.

	Mean ± SD	Median (IQR)	Range
**Age**	10.01 ± 2.552	9 (8–11)	6–17
**SPAS**	7.94 ± 5.268	10 (5–13)	0–23
**PUTS**	17.55 ± 3.461	17 (15–20)	13–31
**YGTSS**	25.39 ± 10.928	23 (16–32)	5–64
** Motor tic**	14.03 ± 4.590	14 (11–17)	0–25
** Vocal tic**	8.40 ± 5.785	8 (3–12)	0–23
** Impairment of tic**	2.69 ± 5.472*	0 (0–0)	0–30
**CY-BOCS**	5.61 ± 6.723*	3 (0–10)	0–27
** Obsessive thought**	2.37 ± 3.705*	0 (0–4)	0–15
** Compulsive behavior**	3.23 ± 4.096*	1 (0–6)	0–16

SPAS, Sensory Phenomenon Assessment Scale; PUTS, Premonitory Urge to Tic Scale; YGTSS, Yale Global Tic Severity Scale; CY-BOCS, Children’s Yale-Brown Obsessive–Compulsive Scale; SD, standard deviation; IQR, interquartile range.

*These data do not follow normal distribution.

### Reliability of SPAS

#### Internal consistency

The Cronbach’s α coefficients for the SPAS were 0.844 for the total sample (*n* = 223), 0.870 for group 1 (aged less than 10 years), and 0.801 for group 2 (aged more than or equal to 10 years). Furthermore, the Spearman–Brown coefficients for these samples were 0.866, 0.901, and 0.813, respectively, indicating strong split-half reliability. For more details, see **Table 2**.

**Table 2:** Reliability of SPAS.

	Younger group (*n* = 124)	Older group (*n* = 99)	Total (*n* = 223)
Cronbach’s alpha	0.870	0.801	0.844
Spearman–Brown	0.901	0.813	0.866

SPAS, Sensory Phenomenon Assessment Scale; Younger group: age < 10; Older group: age ≥ 10.

#### Test–retest reliability

Fifty-three patients were selected and refilled the SPAS 1 month later. The correlation coefficient between the two measurements was 0.987 (*p* < 0.01).

### Validity of SPAS

#### Criterion-related validity

The total SPAS scores of the total, younger, and older groups were significantly positively correlated with scores of the PUTS (*p* < 0.01), but not with the YGTSS (*p* > 0.05) or the CY-BOCS (*p* > 0.05). Each correlation coefficient is shown in **Table 3**. The correlations among each item of the SPAS and the respective scales and subscales are presented in **Supplementary Table S3**. **Supplementary Table S4** displays the correlations among each item of the SPAS.

**Table 3:** Validity of SPAS.

	Younger group (*n* = 124)	Older group (*n* = 99)	Total (*n* = 223)
Criterion-related validity			
PUTS and SPAS	0.578*	0.389*	0.494*
YGTSS and SPAS	−0.050	0.030	−0.027
CY-BOCS and SPAS	−0.047	0.136	0.060
**Bartlett’s test of sphericity**	309.019*	160.231*	464.436*
Factor loading for exploratory factor analysis**			
Item 9	0.831	0.724	0.790
Item 10	0.851	0.832	0.844
Item 11	0.846	0.761	0.814
Item 12	0.838	0.801	0.820
Item 13	0.720	0.608	0.662

PUTS, Premonitory Urge to Tic Scale; SPAS, Sensory Phenomenon Assessment Scale; YGTSS, Yale Global Tic Severity Scale; CY-BOCS, Children’s Yale-Brown Obsessive–Compulsive Scale; *p < 0.01; **Only one factor was analyzed by exploratory factor analysis for each sample, and the variance contributions for all of the three sample rates were 67.024%, 56.162%, and 62.242%.

#### Validity of construct

The score of each item of the SPAS was significantly correlated with the total score. In addition, the EFA with the total sample identified one dimension that explained 62.242% of the variance. Furthermore, each item from both total sample and younger or older sample had a factor loading greater than 0.5. For more details, see **Table 3**.

## Discussion

The present study reports the development and validation of the SPAS, a new tool to quantitatively measure individual differences in tic-related sensory phenomena. Concurring with our hypotheses, the SPAS demonstrated good psychometric properties.

The final version of the SPAS was developed through literature search and Delphi expert consultation. The overall framework of the SPAS appeared to be reasonable for assessing sensory symptoms associated with TD in children and adolescents. Some reasons supporting the reasonableness of the framework were as follows.

On the one hand, the SPAS has been designed to be an observer-rated scale. Previous scales (15, 18, 19) used to assess PU or sensory symptoms were all self-reported, with the exception of I-PUTS (17). The SPAS and I-PUTS were clinician-rated scales, which might avoid the interference of subjectivity from patients. A meta-analysis (30) indicated that clinician-rated instruments commonly enjoy significantly higher effect sizes than their self-reported counterparts, though another study (31) stated that both self-report scales and clinician-rated scales were irreplaceable and complement each other.

On the other hand, the two-part structure of SPAS, with a symptom list and a severity assessment, was a well-thought-out design. The symptom list captured the variety of sensory phenomena associated with TDs, while the severity assessment quantified the intensity and impact of these symptoms on the child’s daily life. The severity assessment part of the SPAS included items such as number, frequency, tensity, degree of transformation, and functional impairment. Given that the I-PUTS evaluated PU only by three dimensions (number, frequency, and intensity), the SPAS may serve as a more comprehensive tool for assessing PU in TD.

The SPAS demonstrated satisfactory performance in terms of these reliability and validity. The 1-month test–retest reliability assessment of SPAS revealed highly significant correlations (*p* < 0.01), with a correlation coefficient of 0.987. Our findings affirm the stability and reliability of the measurement instrument, meeting the prescribed criteria for reliability. There is strong reliability for both the younger population, under 10 years of age, and the older group comprising children and adolescents aged 10 years and above. Furthermore, this observation compensates for the less than satisfactory reliability exhibited by the previous tool PUTS when applied to patients with TS under the age of 10 (32). Evidence suggested that the incidence of PU in patients with TD increases with age (33). The reason for this may involve a growing physiological awareness (or body awareness) with age. This also made older children more aware of the presence of PU. Other studies have suggested that this body awareness was negatively related to inhibitory function (34), and PU seemed to be related to inhibitory function (12); thus, we hypothesized that the incidence of PU increases with age, possibly because of increased body awareness. This also made it difficult for younger children (especially under the age of 10) to understand the meaning of the items when they complete the self-rating questionnaire (because they may never be aware of the intuitive feeling).

Subsequently, the validity analysis had factor loadings greater than 0.5 after EFA, indicating that these items together contribute to one dimension—the severity of PU. The unidimensional scale has demonstrated benefits in clinical settings due to its simplicity for clinical assessors, straightforward administration, and the ease with which results can be shared for clinical reference.

We then selected the PUTS, YGTSS, and CY-BOCS as criteria for calculating criterion validity. The total score of the SPAS exhibited a significant positive correlation with the total score of the PUTS (*p* < 0.001). However, there was no observed correlation between the total score of the SPAS and the total score of the YGTSS and CY-BOCS. These findings suggest that the SPAS demonstrates robust criterion validity, primarily assessing the severity of PU (as indicated by its lack of correlation with the YGTSS and CY-BOCS total score), rather than other measures such as tic symptoms and obsessive–compulsive symptoms. However, in most prior investigations of the PUTS (15), PUTS-R (16), and I-PUTS (17), a least moderate significant correlation with the YGTSS was presented. The reason for our inconsistency with previous results may be that our sample consisted of only patients with TD aged 6–16 years with PU. A larger sample size and more age-stratified patients with TD (such as young adults) may be needed in the future to explore the correlation between the results of the SPAS and other scales.

Our study has several limitations. Firstly, for convenient access, our sample was composed of children and adolescents exclusively. Future investigations should include adults with TD for gaining comprehensive insight. Secondly, while we employed the YGTSS, the PUTS, and the CY-BOCS as concurrent validity measures, alternative scales such as the USP-SPS (20), specifically designed for PU assessment, might offer alternative results. Lastly, we acknowledge that the lack of direct measurement of participants’ intelligence quotient (IQ) is a limitation of our study. While clinicians excluded participants who demonstrated difficulties in communication, which often correlates with lower IQ, we did not administer formal IQ tests as part of our protocol. In future studies, we will exclude the effect of IQ on the results.

## Conclusion

Following psychometric testing, the newly developed scale SPAS demonstrated robust reliability and validity, fulfilling the prerequisites for clinical scales. Subsequent clinical applications confirmed the utility of this scale, indicating its capacity to provide a comprehensive evaluation of PU severity in children and adolescents with TD aged 6–17 years.

## Data availability statement

The raw data supporting the conclusions of this article will be made available by the authors, without undue reservation.

## Ethics statement

We obtained the informed consent of all patients and obtained the ethical approval issued by the Ethics Committee of Beijing Children’s Hospital.

## Author contributions

XW: Writing – review & editing, Writing – original draft, Software, Resources, Project administration, Methodology, Data curation. YLL: Writing – original draft, Software, Resources, Project administration, Investigation, Data curation. LY: Writing – review & editing, Supervision, Methodology. HX: Writing – review & editing, Methodology, Investigation. AZ: Writing – review & editing, Supervision, Methodology, Investigation. WZ: Writing – review & editing, Supervision, Methodology, Investigation. ZJ: Writing – review & editing, Supervision, Methodology, Investigation. YC: Writing – review & editing, Supervision, Funding acquisition, Conceptualization. YL: Writing – review & editing, Supervision, Project administration, Funding acquisition, Conceptualization.
