# Supplementary material for: Sensory Phenomenon Assessment Scale: a new tool for assessment of tic-associated sensations
Source: Front Psychiatry. 2024 Jun 24;15:1387417. doi: 10.3389/fpsyt.2024.1387417 (PMC11228244; doi:10.3389/fpsyt.2024.1387417)
Supplement: Supplementary file 1 [file DataSheet_1.zip › Development of Sensory Phenomenon Assessment Scale (SPAS).DOCX]

# Development of Sensory Phenomenon Assessment Scale

## methods

### Literature review to build items pool

The developing team of SPAS was from the Department of Psychiatry, Beijing Children's Hospital, including one chief physician, two associate chief physicians, two attending physicians, and four postgraduate students. By reviewing the relevant literature on the assessment of PU and tic disorders, the characteristics of PU were understood. Our team members extracted key information of the literature and combined clinical experience of the team to create a new scale. Based on the literature review, main dimensions and initial establishment of items pool were drafted.

### Delphi expert consultation

Several experts in the field of child psychiatry and neuropsychology were invited for Delphi consultation^26^. The inclusion criteria of consultation experts were as followed: (a) has been engaged in child psychiatry and clinical neuropsychology work for at least 15 years; (b) has associate chief title or above; (c) has master’s degree or above; (d) voluntarily participate in the research.

The consultation questionnaires were sent to all experts by email, and were answered within 2 weeks. The first part of questionnaire included the introduction of the research content, form filling instructions, content of items. The second part enquired whether the experts agreed with the items. The importance of the items was evaluated, and the score ranged from 1 to 5, with higher score reflecting higher importance of the items. Experts also put forward their opinions and additions for the entire SPAS. Item's familiarity (Cs) were rated on a Likert scale of 5, ranging from 0.2 (unfamiliar) to 1.0 (very familiar). Four aspects were taken into consideration: theoretical analysis, practical experience, data reference and intuitive feeling (see Table s1). The authoritative coefficient (Cr) was determined by two factors: the judgment basis of the indicators (Ca) and the familiarity with the indicator (Cs). The calculation formula is Cr = (Ca + Cs)/2. The value of Ca and Cs was obtained through self-evaluation of the experts. The larger the Cr value, the higher the authoritative coefficient, and Cr ≥ 0.7 is regarded as the sufficiency threshold.

Items were then selected according to their importance scores, coefficients of variation and full score ratio. The conditions for deleting items included: ① The item importance score < the average score standard deviation of all items; ② The variation coefficient of the importance score of this item > conforms to the average variation coefficient + standard deviation (SD) of all items; ③ The full score ratio of the item≤ the average full score ratio of all items - SD.

## Results

### Items pool and first-round Delphi expert consultation

A total of 62 articles were retrieved. By reading the title and abstract of the literature, the scales related to the assessment of sensory symptoms in patients with tic disorder were selected. After the discussion of the expert group, the articles were screened and summarized on the basis of the comparative theoretical framework. The preliminary entry pool including 6 first-level indicators (dimensions) and 43 second-level indicators (items) were formed. The first-level indicator included location of sensory phenomena, frequency, degree of awareness, level of tension, degree of transformation, impairment of function. Each of the first five dimensions has the following eight items: itch, sense of suffocation, pressure, sense of energy release, sense of tension, sense of uncompletion, indescribable discomfort, other types of sensory symptoms. The sixth dimension has the following three items: Work/study, social and family function. For more details, see **Supplementary Table s2**

A total of 12 questionnaires were issued in the first round of expert consultation, with a recovery rate of 100%. Mean age of the experts was 49.5 ± 4.09 years, with 22.5 ± 4.25 years of clinical practice. The consulting experts involved 6 child psychiatrist, 3 neurologists, 1 psychologist and 2 epidemiologists. In the second round of expert consultation, another 12 questionnaires were issued and recovered. Basic information and authoritative evaluation of 12 experts was shown in Table 1. The result of the importance score for each item according to 12 experts were listed in Table 2.

**Table 1 Basic information and authoritative evaluation of 12 experts**

| **Expert ID** | **Gender** | **Age (years)** | **Work years** | **Degree** | **Title** | **Ca** | **Cs** | **Cr** |
| --- | --- | --- | --- | --- | --- | --- | --- | --- |
| 1 | Male | 54 | 26 | Doctor | Chief psychiatrist | 0.8 | 0.6 | 0.7 |
| 2 | Female | 53 | 25 | Doctor | Chief psychiatrist | 0.9 | 0.8 | 0.85 |
| 3 | Female | 48 | 20 | Doctor | Associate chief psychiatrist | 0.8 | 0.8 | 0.8 |
| 4 | Male | 49 | 22 | Doctor | Chief psychiatrist | 1 | 1 | 1 |
| 5 | Female | 48 | 21 | Doctor | Chief psychiatrist | 0.9 | 0.8 | 0.85 |
| 6 | Male | 56 | 31 | Master | Chief psychiatrist | 0.7 | 0.8 | 0.75 |
| 7 | Female | 55 | 29 | Master | Chief neurologist | 0.8 | 1 | 0.9 |
| 8 | Female | 51 | 23 | Doctor | Chief neurologist | 0.9 | 1 | 0.95 |
| 9 | Male | 47 | 19 | Doctor | Associate chief neurologist | 1 | 0.8 | 0.9 |
| 10 | Female | 45 | 18 | Master | Associate chief psychologist | 0.8 | 0.6 | 0.7 |
| 11 | Female | 45 | 19 | Doctor | Chief epidemiologist | 0.8 | 0.6 | 0.7 |
| 12 | Female | 43 | 17 | Doctor | Associate chief epidemiologist | 0.9 | 0.6 | 0.75 |

**Table 2 The importance scores of each item in the first round of expert consultation questionnaire (n = 12)**

| **Itmes** | **Score range** | **Mean** | **SD** | **Coefficient of variation (%)** | **Full score ratio (%)** |
| --- | --- | --- | --- | --- | --- |
| 1 | 4-5 | 4.833333 | 0.372678 | 7.710579 | 83.33333 |
| 2 | 4-5 | 4.75 | 0.433013 | 9.116057 | 75 |
| 3 | 4-5 | 4.666667 | 0.471405 | 10.10153 | 66.66667 |
| 4 | 4-5 | 4.75 | 0.433013 | 9.116057 | 75 |
| 5 | 4-5 | 4.583333 | 0.493007 | 10.75651 | 58.33333 |
| 6 | 4-5 | 4.75 | 0.433013 | 9.116057 | 75 |
| 7 | 4-5 | 4.666667 | 0.471405 | 10.10153 | 66.66667 |
| 8 | 4-5 | 4.833333 | 0.372678 | 7.710579 | 83.33333 |
| 9 | 4-5 | 4.833333 | 0.372678 | 7.710579 | 83.33333 |
| 10 | 4-5 | 4.833333 | 0.372678 | 7.710579 | 83.33333 |
| 11 | 4-5 | 4.916667 | 0.276385 | 5.621398 | 91.66667 |
| 12 | 4-5 | 4.833333 | 0.372678 | 7.710579 | 83.33333 |
| 13 | 4-5 | 4.75 | 0.433013 | 9.116057 | 75 |
| 14 | 5-5 | 5 | 0 | 0 | 100 |
| 15 | 5-5 | 5 | 0 | 0 | 100 |
| 16 | 5-5 | 5 | 0 | 0 | 100 |
| 17 | 1-4 | 1.833333 | 0.986013 | 53.78254 | 0 |
| 18 | 1-3 | 1.666667 | 0.849837 | 50.9902 | 0 |
| 19 | 1-3 | 1.666667 | 0.849837 | 50.9902 | 0 |
| 20 | 1-3 | 1.666667 | 0.849837 | 50.9902 | 0 |
| 21 | 1-3 | 1.75 | 0.924211 | 52.81208 | 0 |
| 22 | 1-3 | 1.666667 | 0.849837 | 50.9902 | 0 |
| 23 | 1-3 | 1.666667 | 0.849837 | 50.9902 | 0 |
| 24 | 1-3 | 1.583333 | 0.759203 | 47.94965 | 0 |
| 25 | 5-5 | 5 | 0 | 0 | 100 |
| 26 | 4-5 | 4.916667 | 0.276385 | 5.621398 | 91.66667 |
| 27 | 5-5 | 5 | 0 | 0 | 100 |
| 28 | 4-5 | 4.833333 | 0.372678 | 7.710579 | 83.33333 |
| 29 | 4-5 | 4.833333 | 0.372678 | 7.710579 | 83.33333 |
| 30 | 4-5 | 4.916667 | 0.276385 | 5.621398 | 91.66667 |
| 31 | 5-5 | 5 | 0 | 0 | 100 |
| 32 | 5-5 | 5 | 0 | 0 | 100 |
| 33 | 5-5 | 5 | 0 | 0 | 100 |
| 34 | 5-5 | 5 | 0 | 0 | 100 |
| 35 | 5-5 | 5 | 0 | 0 | 100 |
| 36 | 5-5 | 5 | 0 | 0 | 100 |
| 37 | 4-5 | 4.916667 | 0.276385 | 5.621398 | 91.66667 |
| 38 | 5-5 | 5 | 0 | 0 | 100 |
| 39 | 4-5 | 4.916667 | 0.276385 | 5.621398 | 91.66667 |
| 40 | 4-5 | 4.916667 | 0.276385 | 5.621398 | 91.66667 |
| 41 | 5-5 | 5 | 0 | 0 | 100 |
| 42 | 4-5 | 4.833333 | 0.372678 | 7.710579 | 83.33333 |
| 43 | 4-5 | 4.833333 | 0.372678 | 7.710579 | 83.33333 |

### second-round of expert consultation and the final version of the scale

We then analyzed the results from first round of expert consultation. The third dimension “degree of awareness” was deleted due to low importance score. According to the experts, the eight symptoms should not appear under each of the first five dimensions and should be separated to form a "symptom list" dimension. Additionally, the number of sensory symptoms varies from patient to patient, so the "symptom list" can be considered without scoring, just marking the corresponding sensory symptoms and the location of the body that appear. However, as the number of sensory symptoms is an important quantitative indicator, we added "number of sensory symptoms" to the total score.

Based on the comments above, we modified the scale to include 2 parts, namely symptom list and severity of symptom. In the "Symptom List", we made a list of seven common sensory symptoms (itch, sense of suffocation, pressure, sense of energy release, sense of tension, sense of uncompletion, indescribable discomfort). If the subject's sensory symptoms did not fall within these classifications, they could fill in item 8, "Other". This section also allows the investigator to record the location of tics. This section only records the symptoms and the locations, therefore no points are scored. The "symptom severity" consisted of 5 items, namely number, frequency, tensity, degree of transformation and functional impairment. This revised version was made into an expert consultation form (**Supplementary Table s3**) and reviewed by 10 experts in the second round. Experts scored the importance of each item, and the results are presented in **Table 3**.

**Table 3 The importance scores of each item in the second round of expert consultation questionnaire (n = 10)**

| Itmes | Score range | Mean | SD | Coefficient of variation (%) | Full score ratio (%) |
| --- | --- | --- | --- | --- | --- |
| 1 | 4-5 | 4.9 | 0.3 | 6.122449 | 90 |
| 2 | 2-5 | 4 | 1.095445 | 27.38613 | 50 |
| 3 | 4-5 | 4.9 | 0.3 | 6.122449 | 90 |
| 4 | 5-5 | 5 | 0 | 0 | 100 |
| 5 | 4-5 | 4.7 | 0.458258 | 9.750161 | 70 |
| 6 | 5-5 | 5 | 0 | 0 | 100 |
| 7 | 5-5 | 5 | 0 | 0 | 100 |
| 8 | 4-5 | 4.8 | 0.4 | 8.333333 | 80 |
| 9 | 4-5 | 4.9 | 0.3 | 6.122449 | 90 |
| 10 | 5-5 | 5 | 0 | 0 | 100 |
| 11 | 4-5 | 4.8 | 0.4 | 8.333333 | 80 |
| 12 | 5-5 | 5 | 0 | 0 | 100 |
| 13 | 5-5 | 5 | 0 | 0 | 100 |

After two rounds of expert consultation, the final version of the SPAS consisited of two parts with 13 items. The first part was the "symptom list", which contained 8 items (itch, sense of suffocation, pressure, sense of energy release, sense of energy tension, sense of uncompletion, indescribable discomfort, other). The second part is "severity" and contains five items, number, frequency, tensity, degree of transformation and functional impairment. Each item was scored on a six-point scale from 0 to 5, with higher scores indicating severer symptoms. For more details, see **Appendix 2 final version of SPAS** and **Appendix 3 SPAS User Manual**.
