# Supplementary material for: Sensory Phenomenon Assessment Scale: a new tool for assessment of tic-associated sensations
Source: Front Psychiatry. 2024 Jun 24;15:1387417. doi: 10.3389/fpsyt.2024.1387417 (PMC11228244; doi:10.3389/fpsyt.2024.1387417)
Supplement: Supplementary file 1 [file DataSheet_1.zip › Final Version of Sensory Phenomenon Assessment Scale (SPAS).DOCX]

**Final Version of Sensory Phenomenon Assessment Scale (SPAS)**

**Part 1 symptom list**

This part mainly determines the type and location of sensory symptoms. If a single sensory symptom occurs in a single site, it is defined as simple dysesthesia. If there are multiple sensory symptoms in a single site, it is defined as complex abnormal sensation. If none of the above sensory symptoms exist, please finish the evaluation, and the second part of the evaluation should be treated as 0 points. If the first part of the sensory symptoms assessed as present (at least one), then proceed to the second part of the assessment.

Type/location of abnormal sensory symptoms have the following categories? Please mark the location in the corresponding box according to the patient's sensory symptoms in the past week, such as "eyes".

| **Item** | **Common types of sensory symptoms** | **existence** | **inexistence** | **location** |
| --- | --- | --- | --- | --- |
| 1 | itch |  |  |  |
| 2 | Sense of suffocation |  |  |  |
| 3 | pressure |  |  |  |
| 4 | Sense of energy release |  |  |  |
| 5 | tension |  |  |  |
| 6 | Sense of uncompletion |  |  |  |
| 7 | indescribable uncomfort |  |  |  |
| 8 | Other types of sensory symptoms are listed here |  |  |  |

PART 2 Severity

| Scoring criteria (based on the patient's performance in the past week) | score |
| --- | --- |
| 9. Number: How many of these sensory symptoms did the subjects have? |  |
| 0= not present;  1 score = 1 simple sensory phenomenon;  2 points = 2 simple sensory phenomena;  3 points = 3 simple sensory phenomena or 1 complex sensory phenomenon;  4 points = 4 simple sensory phenomena or 2 complex sensory phenomena;  5 points = 5 or more simple sensory phenomena or 3 or more complex sensory phenomena. |  |
| 10. Frequency: How often do these sensory symptoms occur? |  |
| 0 score = No appearance;  1 point = less than half a day;  2 points = a majority of 1 day, or a minority of 2 days;  3 points = a majority of 2 days, or a minority of 3-5 days;  4 points = a majority of 3 to 5 days, or a minority of more than 5 days;  5 points = most of the time over 5 days; |  |
| 11. Tensity: |  |
| 0 score = No appearance;  1 point = slight tensity;  2 points = general tensity;  3 points = moderate intensity;  4 points = relatively strong tensity;  5 points = very strong tensity |  |
| 12. Translation：When the above sensory symptoms occur, to what extent do they translate into tic symptoms? |  |
| 0 score = No appearance;  1 = the feeling of individual symptoms will be converted to twitch;  2 = a small amount of sensory symptoms will be converted to twitch;  3 = half feel symptoms will be converted to twitch;  4 = the feeling of most symptoms will be converted to twitch;  5 = almost all sensory symptoms will convert to twitch. |  |
| 13. Functional impairment：How much damage does the presence of these symptoms cause to the subjects' social functioning? |  |
| 0= no impact, almost no impact on daily life/study/work;  1 = minor impact, has a minor influence on daily life/study/work;  2 = mild impact on daily life/study/work some influence;  3 = part, has a moderate effect on daily life/study/work;  4 = serious impact on daily life/study/work has serious influence;  5 = very serious impact on daily life/study/work have very serious impact. |  |
| The total score is calculated by adding the scores for the five dimensions above. |  |
