# Supplementary material for: Sensory Phenomenon Assessment Scale: a new tool for assessment of tic-associated sensations
Source: Front Psychiatry. 2024 Jun 24;15:1387417. doi: 10.3389/fpsyt.2024.1387417 (PMC11228244; doi:10.3389/fpsyt.2024.1387417)
Supplement: Supplementary file 1 [file DataSheet_1.zip › Table S1 The first round of expert consultation questionnaire (for experts).DOCX]

**Supplementary materials**

**Table s1 the first round of expert consultation questionnaire (for experts)**

Introduction: We aimed to develop a new scale to value Premonitory urge (PU) comprehensively. We have built the items pool as following. Please fill in the form below based on your experience and knowledge.

| **Number** | **item** | | | | | | | | Importance score (1-5) |
| --- | --- | --- | --- | --- | --- | --- | --- | --- | --- |
| **I** | **Location of sensory phenomena: Where are these sensory phenomena located in your body?** | | | | | | | |  |
| 1 | itch | | | | | | | |  |
| 2 | Sense of suffocation | | | | | | | |  |
| 3 | pressure | | | | | | | |  |
| 4 | Sense of energy release | | | | | | | |  |
| 5 | tension | | | | | | | |  |
| 6 | Sense of uncompletion | | | | | | | |  |
| 7 | Undescribable discomfort | | | | | | | |  |
| 8 | Other types of sensory symptoms are listed here | | | | | | | |  |
| **II** | **Frequency: How often are these sensory phenomena?** | | | | | | | |  |
| 9 | itch | | | | | | | |  |
| 10 | Sense of suffocation | | | | | | | |  |
| 11 | pressure | | | | | | | |  |
| 12 | Sense of energy release | | | | | | | |  |
| 13 | tension | | | | | | | |  |
| 14 | Sense of uncompletion | | | | | | | |  |
| 15 | Undescribable discomfort | | | | | | | |  |
| 16 | Other types of sensory symptoms are listed here | | | | | | | |  |
| **III** | **Degree of awareness: How many sensory phenomena can you perceive?** | | | | | | | |  |
| 17 | itch | | | | | | | |  |
| 18 | Sense of suffocation | | | | | | | |  |
| 19 | pressure | | | | | | | |  |
| 20 | Sense of energy release | | | | | | | |  |
| 21 | tension | | | | | | | |  |
| 22 | Sense of uncompletion | | | | | | | |  |
| 23 | Undescribable discomfort | | | | | | | |  |
| 24 | Other types of sensory symptoms are listed here | | | | | | | |  |
| **IV** | **Level of tension: How much tension does this make you feel?** | | | | | | | |  |
| 25 | itch | | | | | | | |  |
| 26 | Sense of suffocation | | | | | | | |  |
| 27 | pressure | | | | | | | |  |
| 28 | Sense of energy release | | | | | | | |  |
| 29 | tension | | | | | | | |  |
| 30 | Sense of uncompletion | | | | | | | |  |
| 31 | Undescribable discomfort | | | | | | | |  |
| 32 | Other types of sensory symptoms are listed here | | | | | | | |  |
| **V** | **Degree of transformation: How many sensory symptoms translate into tics?** | | | | | | | |  |
| 33 | itch | | | | | | | |  |
| 34 | Sense of suffocation | | | | | | | |  |
| 35 | pressure | | | | | | | |  |
| 36 | Sense of energy release | | | | | | | |  |
| 37 | tension | | | | | | | |  |
| 38 | Sense of uncompletion | | | | | | | |  |
| 39 | Undescribable discomfort | | | | | | | |  |
| 40 | Other types of sensory symptoms are listed here | | | | | | | |  |
| **VI** | **Impairment of function: To what extent do sensory phenomena impair your social functioning in the following areas?** | | | | | | | |  |
| 41 | Work/Study | | | | | | | |  |
| 42 | Social | | | | | | | |  |
| 43 | Family function | | | | | | | |  |
| supplementary suggestion: | | | | | | | | | |
| **Please fill in "√" in the box below the corresponding level of judgment basis.** | | | | | | | | | |
| **Basis of judgment** | | | **high** | | **mediate** | | **low** | | |
| Practical experience | | |  | |  | |  | | |
| Theoretical analysis | | |  | |  | |  | | |
| Reference to domestic and foreign data | | |  | |  | |  | | |
| Intuitive feeling | | |  | |  | |  | | |
| **How familiar are you with the subject (PU) of our study? Please fill in "√" in the box below the corresponding level of familiarity.** | | | | | | | | | |
| Unfamiliar | | Casual Acquaintance | | Familiar | | Well-Acquainted | | Intimate | |
|  | |  | |  | |  | |  | |

**Table s2 the second round of expert consultation questionnaire (for experts)**

Introduction: We aimed to develop a new scale to value Premonitory urge (PU) comprehensively. We have re-built the item framework after the first round of expert consultation as following. Please fill in the form below based on your experience and knowledge.

| **Number** | **item** | Importance score (1-5) |
| --- | --- | --- |
| **I** | **Symptom list: Where are these sensory phenomena located in your body?** |  |
| 1 | itch |  |
| 2 | Sense of suffocation |  |
| 3 | pressure |  |
| 4 | Sense of energy release |  |
| 5 | tension |  |
| 6 | Sense of uncompletion |  |
| 7 | Undescribable discomfort |  |
| 8 | Other types of sensory symptoms are listed here |  |
| **II** | **Severity of symptoms** |  |
| 9 | Number: How many of these sensory symptoms did the subjects have? |  |
| 10 | Frequency: How often do these sensory symptoms occur? |  |
| 11 | Tension: How nervous are you when you feel the symptoms? |  |
| 12 | Translation：When the above sensory symptoms occur, to what extent do they translate into tic symptoms? |  |
| 13 | Functional impairment：How much damage does the presence of these symptoms cause to the subjects' social functioning? |  |

**Table s3** Correlations between each item of the SPAS

|  | **SPAS-9** | **SPAS-10** | **SPAS-11** | **SPAS-12** | **SPAS-13** |
| --- | --- | --- | --- | --- | --- |
| **SPAS-9** | 1 |  |  |  |  |
| **SPAS-10** | 0.578** | 1 |  |  |  |
| **SPAS-11** | 0.658** | 0.572** | 1 |  |  |
| **SPAS-12** | 0.547** | 0.717** | 0.553** | 1 |  |
| **SPAS-13** | 0.461** | 0.438** | 0.498** | 0.435** | 1 |

Note: **p* < 0.05; ***p* < 0.01

**Table s4** Correlations between each item of the SPAS and the individual scales and subscales

|  | **SPAS-9** | **SPAS-10** | **SPAS-11** | **SPAS-12** | **SPAS-13** |
| --- | --- | --- | --- | --- | --- |
| **YGTSS** | -0.074 | -0.040 | -0.106 | -0.018 | 0.253** |
| motor tic | -0.015 | -0.021 | -0.092 | -0.007 | 0.192** |
| vocal tic | -0.157* | -0.135* | -0.206** | -0.129 | 0.010 |
| Impairment of tic | 0.109 | 0.133* | 0.157* | 0.147* | 0.443** |
| **PUTS** | 0.462** | 0.413** | 0.360** | 0.410** | 0.302** |
| **CY-BOCS** | 0.022 | 0.071 | 0.027 | 0.024 | 0.165* |
| obsessive thoughts | 0.084 | 0.194** | 0.095 | 0.110 | 0.234** |
| Compulsive behavior | -0.019 | -0.005 | 0.015 | -0.031 | 0.098 |

Note: **p* < 0.05; ***p* < 0.01


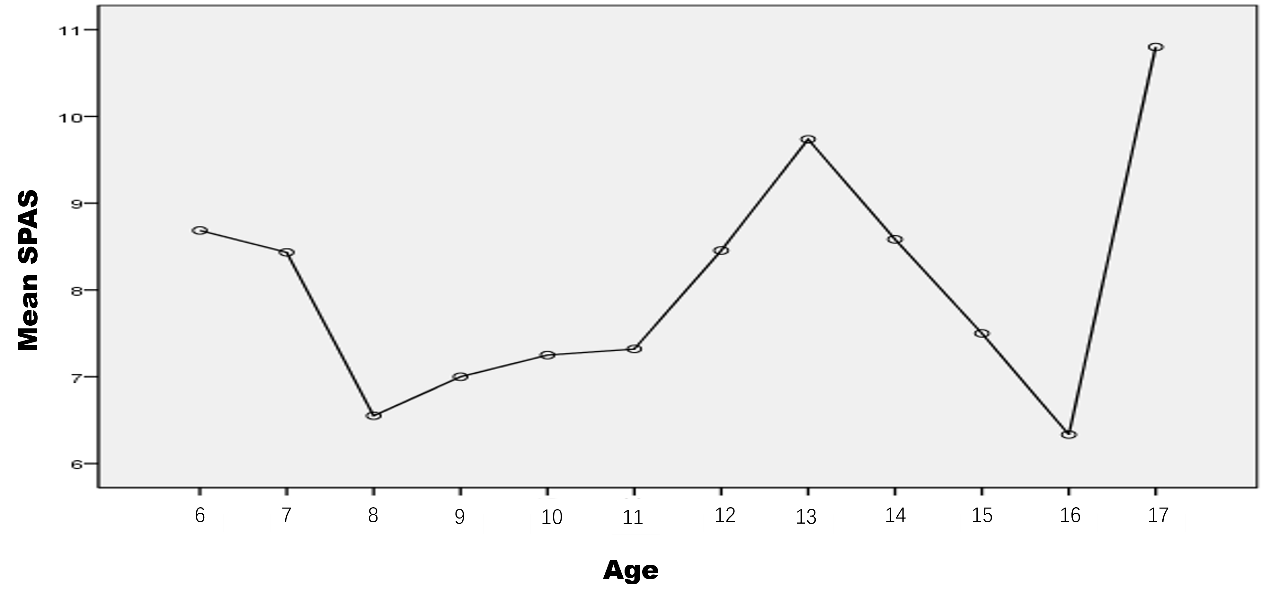


**Fig. s1** Line plot of mean SPAS with age


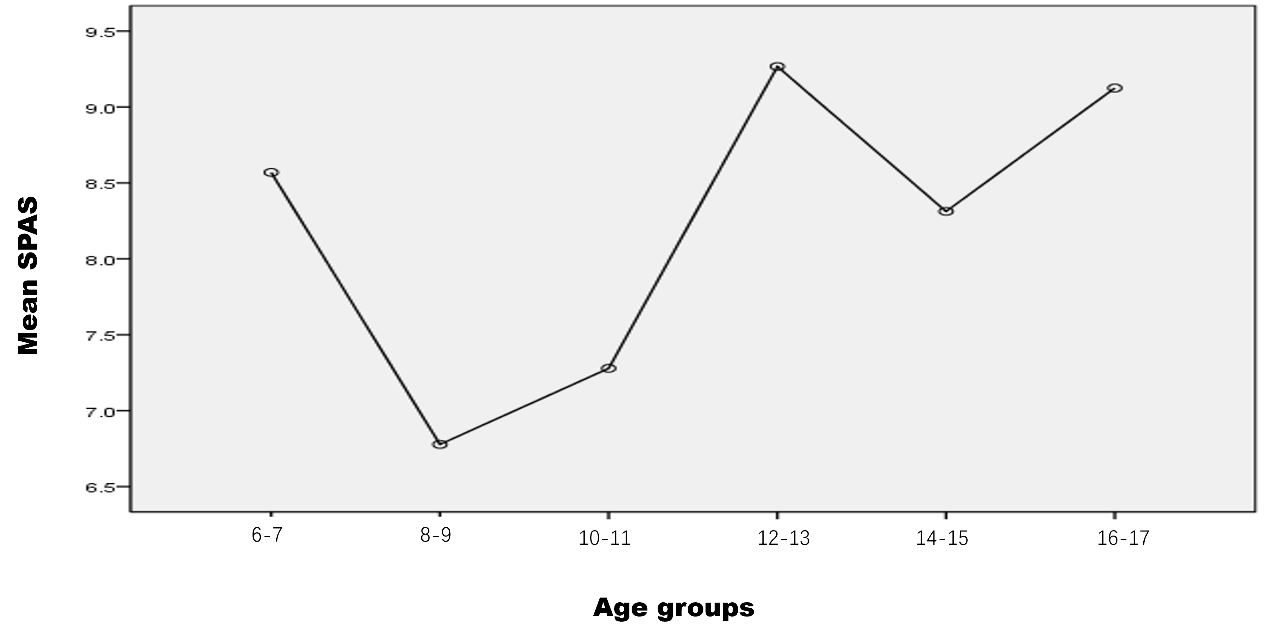


**Fig. s2** Line plot of mean SPAS with age groups


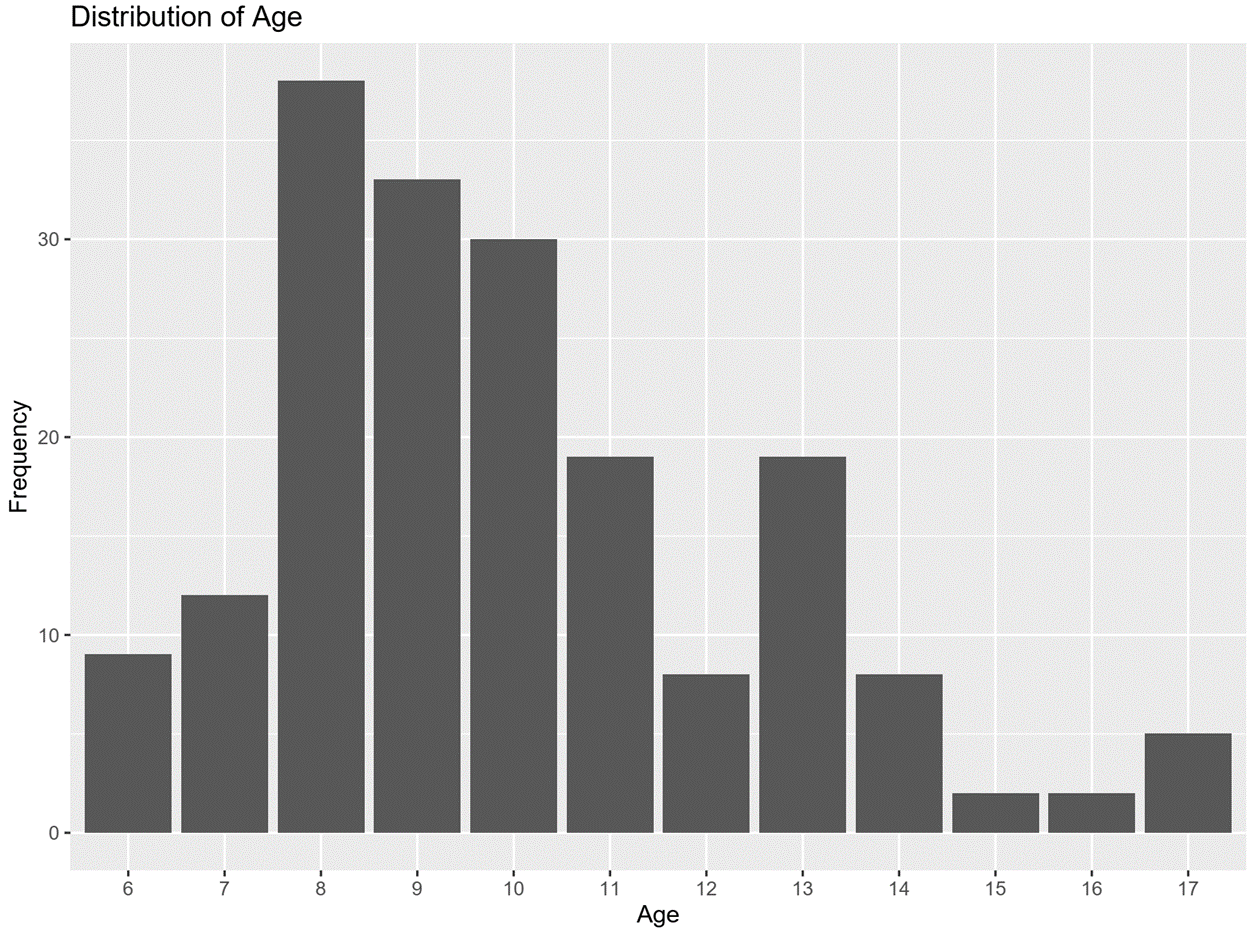


Fig s3 Age distribution of participants
