# Supplementary material for: Sensory Phenomenon Assessment Scale: a new tool for assessment of tic-associated sensations
Source: Front Psychiatry. 2024 Jun 24;15:1387417. doi: 10.3389/fpsyt.2024.1387417 (PMC11228244; doi:10.3389/fpsyt.2024.1387417)
Supplement: Supplementary file 1 [file DataSheet_1.zip › User Manual of Sensory Phenomenon Assessment Scale (SPAS).docx]

**User Manual of Sensory Phenomenon Assessment Scale (SPAS)**

Department of Psychiatry, Beijing Children’s Hospital, Capital Medical University

**Introduction of The Sensation Phenomena Assessment Scale (SPAS)**

The SPAS evaluates a particular sensory phenomenon associated with tics, known as the Premonitory Urge (PU). PU encompasses discomforting bodily sensations that precede tics, with two recognized dimensions: sensory aspects like "itching" or "pressure" in specific body areas, and emotional dimensions, such as the sensation of something being "incomplete."

The SPAS is a thorough evaluation tool crafted by the Psychiatry Department at Beijing Children's Hospital, Capital Medical University. It was meticulously developed through a multifaceted process, incorporating literature research, semi-structured interviews, expert consultations using the Delphi method. SPAS consists of scales that provide a comprehensive assessment of PU across five items: quantity, frequency, intensity, transformation degree, and functional impairment. Professionals may utilize this assessment to tap into the patient's tic symptoms over the previous week.

The interview should ensure that both children and parents (or caregivers) participated in the interviews together. This approach allowed for effective communication, especially among younger participants who may have had difficulty comprehending certain questions independently. Parents (or caregivers) were present to assist their children in understanding and responding appropriately to the interview questions, thereby mitigating potential language comprehension challenges.

Diverging from typical premonitory urge scales that primarily address symptom severity, SPAS offers a dual structure, comprising a symptom list and a severity assessment. The symptom list straightforwardly enumerates the particular sensory phenomena and their locations as reported by the patient. The table below provides a summary of the items and scoring criteria for both components.

**Part 1 symptom list**

This section primarily focuses on identifying the types and locations of sensory symptoms. A single sensory symptom occurring at one location is categorized as a simple sensory anomaly. If multiple sensory symptoms occur at one location, it is considered a complex sensory anomaly. If none of the mentioned sensory symptoms are present, the assessment concludes, and the score for the second part is noted as 0. The assessment may progresses to the second part provided that any sensory symptom was identified in the first part (at least one type).

| **Item** | **Title** | **Evaluation basis** |
| --- | --- | --- |
| 1 | itch | Whether the patient has an itching sensation |
| 2 | Sense of suffocation | Whether the patient has a feeling of suffocation |
| 3 | pressure | Whether the patient has a feeling of being pressed |
| 4 | Sense of energy release | If the patient has a feeling that there is a burst of energy in the body, and whether the twitch serve as a release of this energy |
| 5 | Sense of tension | Whether the patient is worried about the emergence of impulse, resulting in anticipatory anxiety and tension |
| 6 | Sense of uncompletion | Does the patient appear to have a feeling that something is not being done |
| 7 | Indescribable discomfort | Whether the patient has experienced indescribable discomfort |
| 8 | Other types of sensory symptoms are listed here | Whether the patient has any other sensory symptoms |
| none ☐  there are（ ）simply abnormal sensory ☐  there are（ ）complex abnormal sensory ☐ | | |

PART 2 Severity

| **Item** | **Title** | **Evaluation basis** |
| --- | --- | --- |
| 9 | Number | The number of sensory symptoms reported by patients, i.e. how many sensory symptoms appeared |
| 10 | frequency | The frequency of the above sensory symptoms reported by the patient during the week |
| 11 | tensity | Patient-reported concern about sensory symptoms and fear of progression |
| 12 | Transformation | The frequency of the patient's reported impulse translated into tics |
| 13 | Functional impairment | The extent to which patient-reported sensory symptoms impair the individual’s academic, work, social and other functions |

Every assessment relies on semi-structured interviews featuring prompts and inquiries. Entries are evaluated using a 6-point scale (0-5), each score corresponds to a range from no symptoms (0) to severe symptoms (5). Following are the semi-structured interview scripts and comprehensive ratings for each entry in Part II:

**Semi-structured interview script**

**Opening line:**

Hello young friend! Let's finish a quiz together, OK? I'd like to know something about you. Have you been feeling under the weather lately?

**Evaluation of the first part:**

Were you aware of it before it occurred, or did you sense that you were about to twitch in the next moment? Can you provide more details about these sensations? (If the interviewee can't provide specific descriptions, inquire about the sensory phenomena listed in items 1-8: Do you experience itching? Stress in your head or body? Nervousness? Feeling unwell? A sense of unfinished business? Like a force leaving your body? If applicable, please specify the area where you experience these sensations.)

Complete the initial part of the symptom list based on the above interview. **Evaluation of the second part**

**9. Quantitative dimension: Evaluate according to the numbers in the first part.**

0= not present;

1 score = 1 simple sensory phenomenon;

2 points = 2 simple sensory phenomena;

3 points = 3 simple sensory phenomena or 1 complex sensory phenomenon;

4 points = 4 simple sensory phenomena or 2 complex sensory phenomena;

5 points = 5 or more simple sensory phenomena or 3 or more complex sensory phenomena.

Notes: According to comments from experts, “simple sensory phenomena” were defined as a single sensory symptom in a single location; “complex sensory phenomena” was defined as multiple sensory in a single location. For example, a person who has only one itching sensation in the back of the neck is considered as a simple sensory phenomenon. Whereas the person also has tension in the right eye, this is noted as another simple sensory phenomenon. But when the person's nose feels itchy and has indescribable discomfort at the same time, this experience is noted as a complex sensory phenomenon.

**10. Frequency dimension: Does the feeling you just mentioned (if there are multiple ones to ask separately) occur every day? Most of the time or occasionally?**

0 score = No appearance;

1 point = less than half a day;

2 points = more than 1 day, or less than 2 days;

3 points = more than 2 days, or less than 3 days;

4 points = 3 to 5 days, or more than 5 days;

5 points = most of the time over 5 days;

**11. Tensity: Do you feel particularly worried before each time you have this feeling? Were you nervous and afraid, always worried about the appearance of this feeling?**

0 score = No symptom;

1 point = slight tensity: when thinking of the negative experience brought about by this uncomfortable feeling, there will be occasional worry, but no physical anxiety such as sweating, heartbeat racing, or suffocation, symptoms does not affect daily function.

2 points = average tensity: During about half the time of the day, the individual was immersed in worry about tic-related feelings, and constantly think about these uncomfortable experiences. However, this worrying mood can be diverted by other things and pause. No somatic symptoms present.

3 points = moderate intensity: During more than half of the time each day, the individual was excessively worried about the uncomfortable experience of sensory symptoms, and this worry is not easily diverted. Occasionally there were restlessness and somatic symptoms such as palpitations, shaking hands, sweating, and frequent urination. Symptoms slightly affects learning and work efficiency, but the individual can still cope.

4 points = relatively strong tensity: During almost all the time every day, the individual was immersed in the uncomfortable experience of worrying about tic symptoms. This worrying mood cannot be removed by diverting attention. Somatic symptoms such as restlessness, palpitations, shaking hands, sweating, and frequent urination often occur. Symptoms affects learning and work efficiency, but the individual can cope with effort.

5 points = very strong tensity: During almost all the time every day, the individual was immersed in the uncomfortable experience of worrying about tic symptoms. This worrying mood cannot be removed by diverting attention. Somatic symptoms such as restlessness, palpitations, shaking hands, sweating, and frequent urination always occur. Symptoms seriously affect the study, work and life of the individual, who is completely unable to cope.

**12. Degree of transformation: how many impulses will be converted into tics. For example, if you have 10 impulses a day, how many of them actually make you tic?**

0 points = no symptom;

1 point = Small amount (0-20%) sensory symptoms will translate into tics;

2 points = Moderate amount (21-49%) of sensory symptoms will translate into tics;

3 points = Half (50%) of sensory symptoms will translate into tics;

4 points = Most (51-79%) of sensory symptoms will translate into tics;

5 points = Almost all (80-100%) sensory symptoms will translate into tics.

**13. Functional impairment: How much damage will the appearance of the above symptoms cause to your social functioning?**

0 = no effect, almost no impact on daily life/study/work.

1 = slight impact, with a slight impact on daily life/study/work. Daily life: The individual may require more time to complete daily tasks or need extra help to complete certain activities; Learning: The individual may require more time to complete assignments or lessons, or need additional tutoring to understand course content; Work: The individual may require more time to complete work tasks, or need extra support to complete certain tasks.

2 = mild effect, some impact on daily life/study/work. Daily life: The individual may require extra help with certain tasks or need additional resources to support daily life; Learning: The individual may require additional tutoring to complete assignments or classes, or need additional resources to support the learning process; Work: The individual may require additional support to complete work tasks, or need additional resources to support the work process.

3 = Partial impact, moderate impact on daily life/study/work. Daily living: The individual may result in the inability to complete certain tasks or activities, or require significant additional support to maintain daily life; Learning: The individual may be unable to complete assignments or courses, or require significant additional resources to support the learning process; Work: The individual may be unable to complete work tasks or require significant additional resources to support the work process.

4 = Serious impact, with a relatively serious impact on daily life/study/work. Daily living: The individual may be unable to maintain basic daily routines or require a lot of extra support to maintain daily routines; Learning: The individual may be unable to complete school, or require significant additional resources to support the learning process; Work: The individual may be unable to complete work, or require a lot of additional resources to support the work process.

5 = Very serious impact, very serious impact on daily life/study/work. Daily living: The individual may be unable to maintain basic daily life or require significant additional support to sustain vital activities; Learning: The individual may be unable to continue schooling or requiring significant additional resources to support basic learning activities; Work: The individual may be unable to continue working, or require significant additional resources to support basic work activities. This can have a significant negative impact on the patient's quality of life and mental health.

The total score is the accumulation of the above 5 items’ scores. Higher score indicates severer level of PUs.

At present, the cut-off value of the SPAS has not been identified.
